# Entrapment of a J-tip guidewire within the pulmonary vein during FARAPULSE^TM^ pulsed field ablation: a case report

**DOI:** 10.1093/ehjcr/ytag503

**Published:** 2026-07-11

**Authors:** Ken Watanabe, Takanori Arimoto, Yuta Kobayashi, Naoaki Hashimoto, Masafumi Watanabe

**Affiliations:** Department of Cardiology, Pulmonology, and Nephrology, Yamagata University School of Medicine, 2-2-2 Iida-Nishi, Yamagata 990-9585, Japan; Department of Cardiology, Pulmonology, and Nephrology, Yamagata University School of Medicine, 2-2-2 Iida-Nishi, Yamagata 990-9585, Japan; Department of Cardiology, Pulmonology, and Nephrology, Yamagata University School of Medicine, 2-2-2 Iida-Nishi, Yamagata 990-9585, Japan; Department of Cardiology, Pulmonology, and Nephrology, Yamagata University School of Medicine, 2-2-2 Iida-Nishi, Yamagata 990-9585, Japan; Department of Cardiology, Pulmonology, and Nephrology, Yamagata University School of Medicine, 2-2-2 Iida-Nishi, Yamagata 990-9585, Japan

**Keywords:** Atrial fibrillation, Pulsed field ablation, Guidewire entrapment, Case report

## Abstract

**Background:**

Pulsed field ablation (PFA) is a rapidly adopted technology for atrial fibrillation (AF) ablation and is associated with a favourable safety profile and a low risk of complications. However, mechanical complications have rarely been reported.

**Case summary:**

A 37-year-old Asian male with paroxysmal AF underwent PFA using the FARAPULSE^TM^ system. Following successful ablation of the left superior pulmonary vein (PV), the J-tip guidewire advanced into the left inferior PV and became completely entrapped. Venography demonstrated that, although the guidewire tip remained intravascular, a proximal segment appeared to course along the vessel wall, suggesting passage through an intraluminal structure. Multiple attempts at withdrawal using push-pull manoeuvres were unsuccessful. Repositioning with a radiofrequency ablation catheter also failed to release the entrapment. Given the risk of vessel rupture and major haemorrhage, the procedure was converted to general anaesthesia in a hybrid operating room with surgical standby. Bridge occlusion balloon-assisted extraction successfully released the entrapped guidewire without vascular injury. The retrieved guidewire had adherent fibrous white tissue, suggesting entrapment by a membranous pulmonary venous structure.

**Discussion:**

Balloon-assisted endovascular retrieval may represent a safe and less invasive alternative to surgical intervention for managing such entrapments. Operators should be aware of this rare but potentially hazardous complication.

Learning points

- Guidewire entrapment within the pulmonary vein is a rare but potentially fatal complication during pulsed field ablation.
- A balloon-assisted extraction strategy may enhance haemostatic safety and help avoid the need for invasive surgical retrieval.

## Introduction

Pulsed field ablation (PFA) is a rapidly adopted non-thermal energy modality for the treatment of atrial fibrillation (AF), providing safe and selective myocardial ablation through irreversible electroporation.^1^ Current clinical guidelines now recommend catheter ablation as a first-line rhythm control option for paroxysmal AF,^2^ leading to a global increase in PFA procedures. The FARAPULSE^TM^ system (Boston Scientific, Marlborough, MA, USA) has demonstrated excellent safety and efficacy for AF ablation in large-scale registries.^3^ The overall rate of major adverse events is low, with only infrequent occurrences of complications such as pericardial tamponade, vascular injury, coronary artery spasm, and stroke. Notably, while cases of oesophageal complications and pulmonary vein (PV) stenosis have not been reported, rare instances of persistent phrenic nerve palsy have been documented in real-world clinical use. Furthermore, as this technology becomes more widely utilized, rare procedural complications such as guidewire entrapment within the PV have recently emerged, with optimal management strategies yet to be established.

## Summary figure

**Figure:**
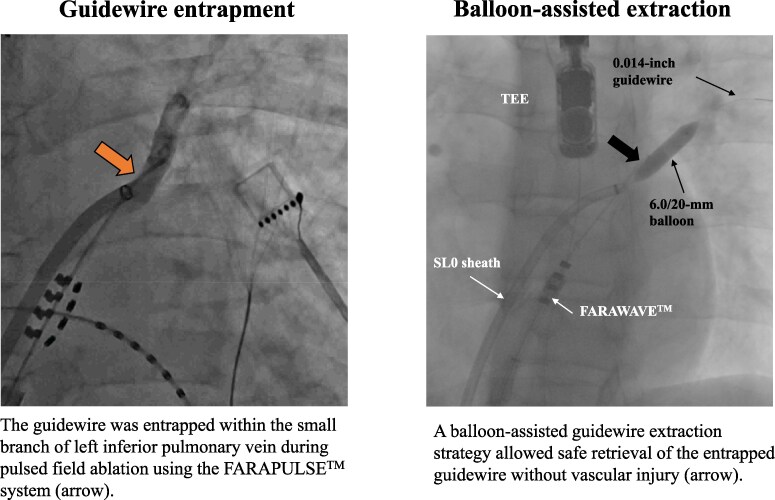


## Case presentation

**Figure 1:**
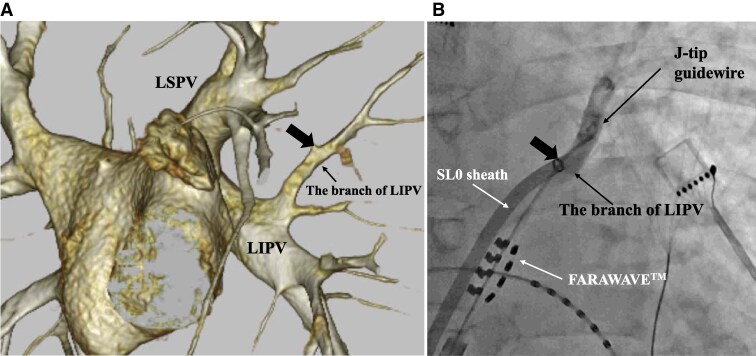
(*A*) Three-dimensional computed tomography showing the left inferior pulmonary vein, where the guidewire subsequently became entrapped (arrow). (*B*) Selective venography showing the J-tip guidewire entrapment within the small branch of the left inferior pulmonary vein (arrow). LIPV, left inferior pulmonary vein; LSPV, left superior pulmonary vein.

A 37-year-old Asian man with no prior medical history or structural heart disease was admitted for PFA using the FARAPULSE^TM^ system to treat symptomatic AF. Pre-procedural transthoracic echocardiography and computed tomography (CT) demonstrated no left atrial enlargement, preserved left ventricular systolic function, absence of left atrial thrombus, and no anatomical abnormalities. Under deep sedation with midazolam and a dexmedetomidine infusion in combination with fentanyl, PV isolation was performed by an experienced electrophysiologist (14 years of clinical experience). Non–invasive positive pressure ventilation was used throughout the procedure. Under intracardiac echocardiographic guidance, transseptal puncture was performed using the VersaCross Connect^TM^ system (Baylis Medical, Montreal, Canada) inserted through the FARADRIVE^TM^ steerable sheath (Boston Scientific). A 12F multi-electrode pentaspline PFA catheter (FARAWAVE^TM^, Boston Scientific) was advanced into the left atrium over a conventional J-tip 0.035-inch guidewire (STARTER^TM^, Boston Scientific). After successful energy delivery in the left superior PV, the operator advanced the guidewire straightforwardly into the left inferior PV without excessive torque or unusual angulation. The guidewire suddenly became immobile, and both advancement and withdrawal were impossible. The patient remained haemodynamically stable, and intracardiac echocardiography showed no pericardial effusion. Selective venography via the SL0 sheath (Abbott Medical, Minneapolis, MN, USA) demonstrated that the distal tip of the guidewire was intravascular, whereas the proximal segment appeared to track along the vessel wall, suggesting partial passage through a membranous structure or sub-intimal tracking (*Figure 1A* and *B*). Attempts to reposition or retrieve the entrapped guidewire by gently manoeuvring an ablation catheter (QDOT Micro, Biosense Webster, Inc., Diamond Bar, CA, USA) alongside it were unsuccessful.

**Figure 2:**
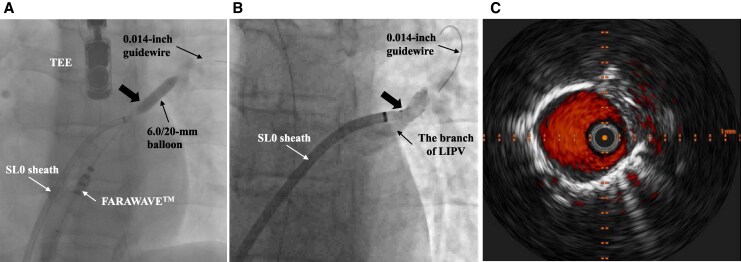
(*A*) Balloon-assisted guidewire extraction at the site of entrapment (arrow) in the left inferior pulmonary vein. A 6.0/20-mm balloon was inflated adjacent to the entrapped segment of the guidewire to stabilize the vessel wall and mitigate potential vascular injury, while the guidewire was carefully withdrawn under continuous transoesophageal echocardiographic monitoring in a hybrid operating room under general anaesthesia. (*B*) Venography confirmed successful guidewire extraction without evidence of vascular injury (arrow). (*C*) Intravascular ultrasound demonstrated an intact pulmonary venous lumen at the site of prior entrapment. LIPV, left inferior pulmonary vein.

**Figure 3:**
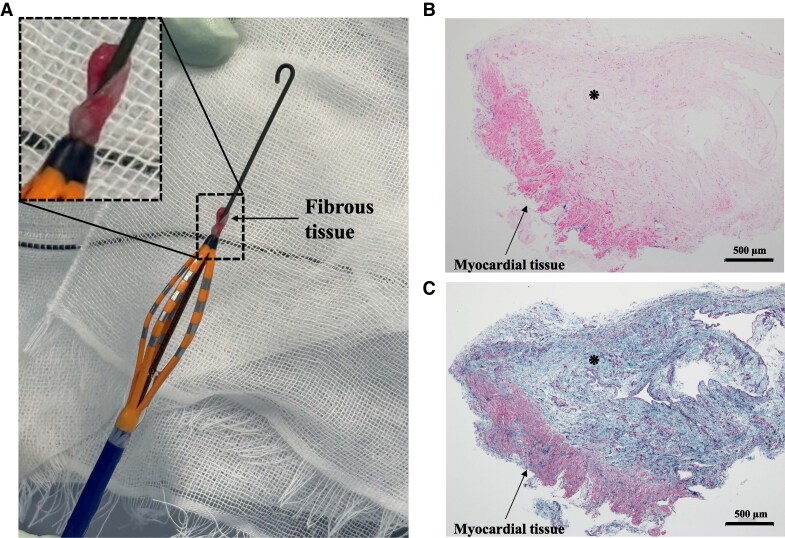
(*A*) Macroscopic examination of the retrieved guidewire demonstrated white fibrous tissue adherent to its surface. The dashed box indicates the region shown at higher magnification. (*B*) Haematoxylin–eosin staining and (*C*) Elastica–Masson staining showed fibrous tissue containing myocardial components. The asterisk (*) indicates connective tissue containing elastic fibres.

Given the risk of vessel rupture and massive haemorrhage with forceful extraction, the procedure was converted to general anaesthesia in a hybrid operating room. A transoesophageal echocardiography (TEE) probe was inserted to provide continuous monitoring during the bailout procedure. A 6.0/20-mm balloon (SHIDEN, Kaneka Medix, Osaka, Japan) mounted on a 0.014-inch Cruise guidewire (Asahi Intecc, Nagoya, Japan) was advanced through the SL0 sheath and inflated to occlude the entrapment site (*Figure 2A*). Under continuous balloon inflation, manual traction was applied to the J-tip guidewire. This manoeuvre successfully released the entrapped guidewire from the left inferior PV. Subsequently, the released guidewire and the FARAWAVE^TM^ catheter were simultaneously withdrawn from the body. The extraction was completed without any evidence of vascular injury or contrast extravasation (*Figure 2B*). Post-retrieval intravascular ultrasound demonstrated a smooth venous lumen without evidence of dissection, residual structures, or thrombus (*Figure 2C*). Transoesophageal echocardiography confirmed the absence of pericardial effusion or haemothorax. Macroscopic inspection of the retrieved guidewire revealed adherent fibrous white material consistent with membranous tissue from the PV (*Figure 3A*). Histopathological examination demonstrated connective tissue with elastic fibres containing myocardial elements (*Figure 3B* and *C*).

A contrast-enhanced CT scan was performed on postoperative Day 1. After confirming the absence of bleeding or vascular injury, anticoagulation therapy was restarted on postoperative Day 2. The patient was discharged 3 days after the procedure without any complications. The patient’s clinical course has been favourable. Despite PFA delivery solely to the left superior PV, the patient has remained AF-free at 1 year, confirmed by electrocardiography and continuous wearable device monitoring. Furthermore, despite the absence of routine contrast-enhanced CT imaging in the chronic phase, the patient’s asymptomatic status suggests that clinically significant late PV stenosis has not occurred.

## Discussion

The FARAPULSE^TM^ system is currently the most widely used PFA device worldwide, supported by robust evidence from both randomized trials and real-world registries demonstrating its safety and efficacy.^1,3–5^ Recently, isolated cases of device and guidewire entrapment associated with PFA systems have been described.^6^ Notably, a case of guidewire entrapment specifically involving the FARAPULSE^TM^ system was reported, in which successful retrieval was achieved using gentle traction. In addition to these cases, several case reports have described an ablation catheter trapped within PV branches necessitating open-heart surgical removal, likely because the branches were small and similar in diameter to the catheter tip.^7,8^ Iwasa *et al*.^9^ reported a case of PulseSelect^TM^ (Medtronic, Minneapolis, MN, USA) guidewire and catheter entrapment within the right superior PV, which was successfully retrieved through the PFA sheath without surgery. They suggested that entrapment occurred when soft PV tissue became trapped in the small gap between the exit portion of the PulseSelect^TM^ catheter and the guidewire. In another report, Furuyama *et al*.^10^ described a case of J-tip guidewire entrapment following isolation of the right inferior PV using the PulseSelect^TM^ system. The guidewire was successfully removed using a conservative approach; however, PV dissection was noted on post-extraction venography. The authors proposed that guidewire manipulation caused PV dissection, leading to entrapment of the guidewire tip. Despite these emerging reports, the exact anatomical mechanisms and optimal, complication-free bailout strategies for guidewire entrapment remain actively discussed. Here, we presented a case of conventional J-tip guidewire entrapment within the PV during FARAPULSE^TM^ PFA procedure, which was successfully managed with a novel balloon-assisted endovascular bailout strategy without vascular injury or the need for surgical intervention.

In the present case, venography demonstrated that the PV lumen was sufficiently large to accommodate the J-tip guidewire, and that there was no evidence of venous dissection. Histological examination of the fibrous tissue adherent to the retrieved guidewire revealed connective tissue with elastic fibres, lacking the major structural components of the vascular wall. These findings suggest that the guidewire traversed a membranous structure within the PV wall, rather than being trapped within a small branch or as a consequence of vascular dissection. Anomalous intracardiac membranous structures, such as the Thebesian valve and the Chiari network, are well-recognized embryological remnants in the right atrium.^11,12^ However, while several developmental anomalies, such as branching anomalies, common PV remnants, and anomalous pulmonary venous connections, are described,^13,14^ there are no established reports of intraluminal membranous structures within the PV. Although the precise mechanisms cannot be definitively established, this case indicates that rare anatomical variants or membranous structures within the PV may predispose to guidewire entrapment during PFA.

Pre-procedural contrast-enhanced CT protocols failed to identify membranous structures, as they likely exist extremely thin, two-dimensional planar networks, falling below the spatial resolution of conventional CT. Given the difficulty of predicting such anatomical variants preoperatively, this complication can occur unexpectedly. As noted in our case, the entrapment occurred despite straightforward guidewire passage without excessive torque by an experienced operator. Therefore, operators should consider this potentially hazardous complication and exercise caution when advancing the guidewire.

When guidewire entrapment occurs, surgical removal is highly invasive. In this case, we adopted a balloon-assisted extraction strategy inspired by the use of bridge occlusion balloons for managing superior vena cava tears during transvenous lead extraction.^15,16^ The balloon sizing was determined based on the diameter of the SL0 sheath and the PV dimensions estimated from intra-procedural venography. This balloon-assisted approach served a dual mechanistic purpose: first, continuous balloon inflation stabilized the surrounding anatomy, facilitating the mechanical release of the guidewire via simple manual traction; second, it acted as a prophylactic occlusion balloon, which enabled immediate haemostatic control in the event of catastrophic bleeding during extraction. This approach contributed to the safe retrieval of the guidewire while mitigating the risk of vessel injury. This balloon-assisted extraction strategy may represent a reasonable, less invasive bailout option before proceeding to thoracotomy or open-heart surgery.

## Conclusion

We describe the case of balloon-assisted extraction of entrapped guidewire during the FARAPULSE^TM^ PFA. The guidewire was successfully retrieved using a balloon-assisted extraction technique under general anaesthesia, without evidence of vascular injury. Awareness and early recognition of this rare but potentially serious complication are essential to enable timely and appropriate management.

## Patient perspective

The patient expressed profound gratitude that the complication was managed safely via an endovascular approach without the need for open-heart surgery, and was satisfied with the seamless recovery without any lasting effects.

## Data Availability

The data underlying this article will be made available by the corresponding author upon reasonable request.

## Lead author biography

Dr Ken Watanabe earned his MD and PhD from Yamagata University School of Medicine. He is currently a cardiologist in the Department of Cardiology, Pulmonology, and Nephrology at the same institution. His primary clinical and research interests focus on catheter ablation for cardiac arrhythmias.

## Notes

**Consent:** The authors confirm that written informed consent for submission and publication of this clinical case report was obtained from the patient in accordance with COPE guidance.
